# Pagetoid dyskeratosis on the abdomen following abdominoplasty: A unique presentation and response to treatment

**DOI:** 10.1016/j.jdcr.2026.09.020

**Published:** 2026-09-20

**Authors:** Shira Wolpowitz, Bisma Khalid, Maya Farah

**Affiliations:** a Department of Dermatology, Newton Wellesley Hospital, Newton, Massachusetts; b Department of Dermatology, Boston University School of Medicine, Boston, Massachusetts; c Assistant Professor of Dermatology, Boston University School of Medicine, Boston, Massachusetts

**Keywords:** abdomen, abdominoplasty, cryotherapy, dyskeratosis, epidermis, incidental, pagetoid dyskeratosis, trauma

## Case presentation

A 45-year-old female with a history of psoriasis presented with asymptomatic lesions on the midline of her abdomen, 9 months after undergoing an uncomplicated abdominoplasty with identification of diastasis recti, which was repaired above and below the umbilicus using a layer of interrupted figure of 8 buried sutures followed by running sutures (Fig 1, *A* and *B*). While these lesions mimic anatomically the suture placement for the diastasis recti repair, the sutures themselves are placed at the level of anterior abdominal wall beneath subcutaneous fat, resulting in no clinical or histologic scar formation in the overlying epidermis or dermis (see Fig 2). She denied any external trauma or friction to the site, or use of postsurgical girdles or compression garments.

**Fig 1:**
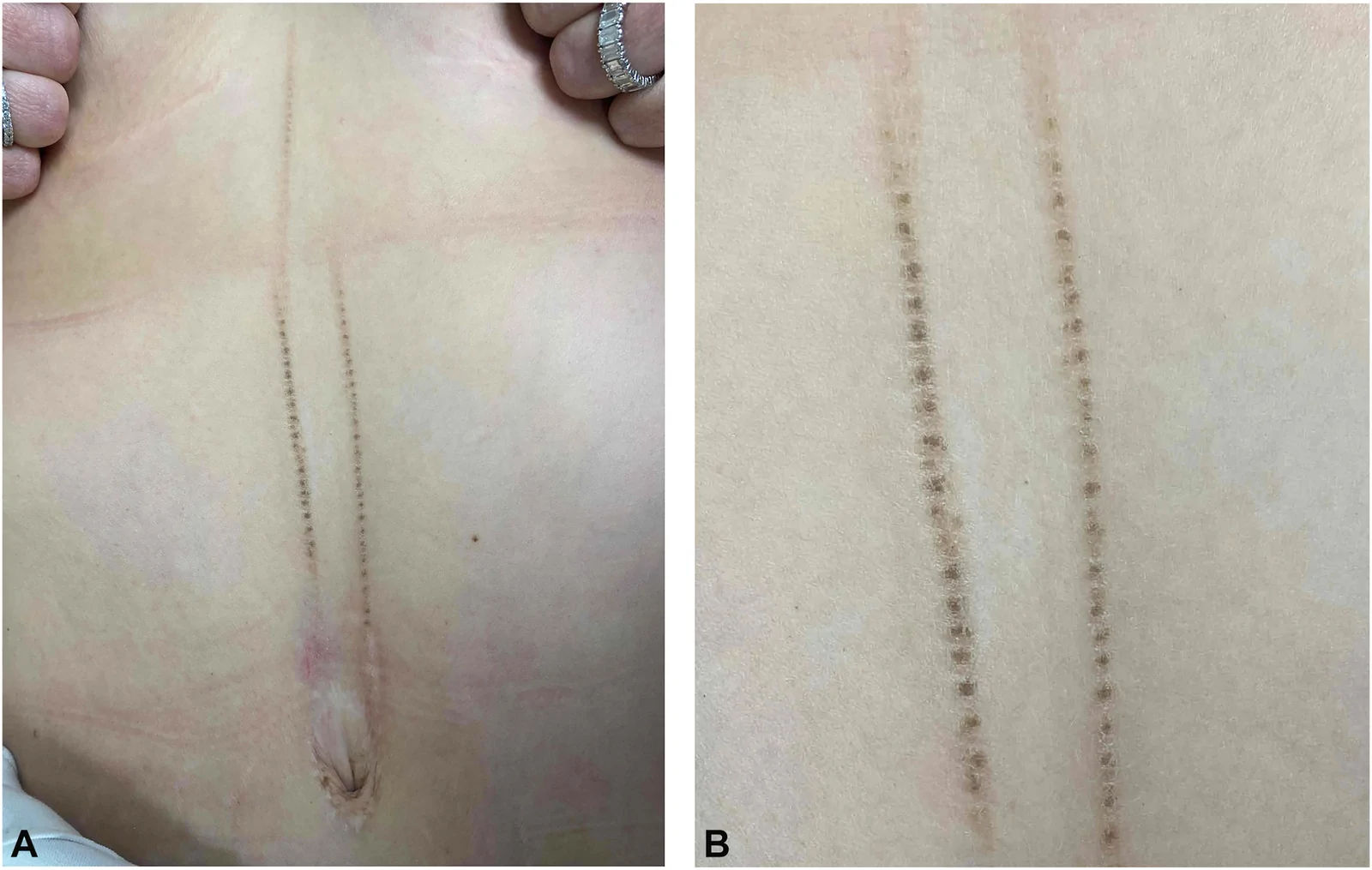
**A,** Clinical examination shows 2 linear brown patches on the midline of the abdomen. **B,** Each patch contains evenly spaced, mildly hyperkeratotic papules in a linear distribution.

**Fig 2:**
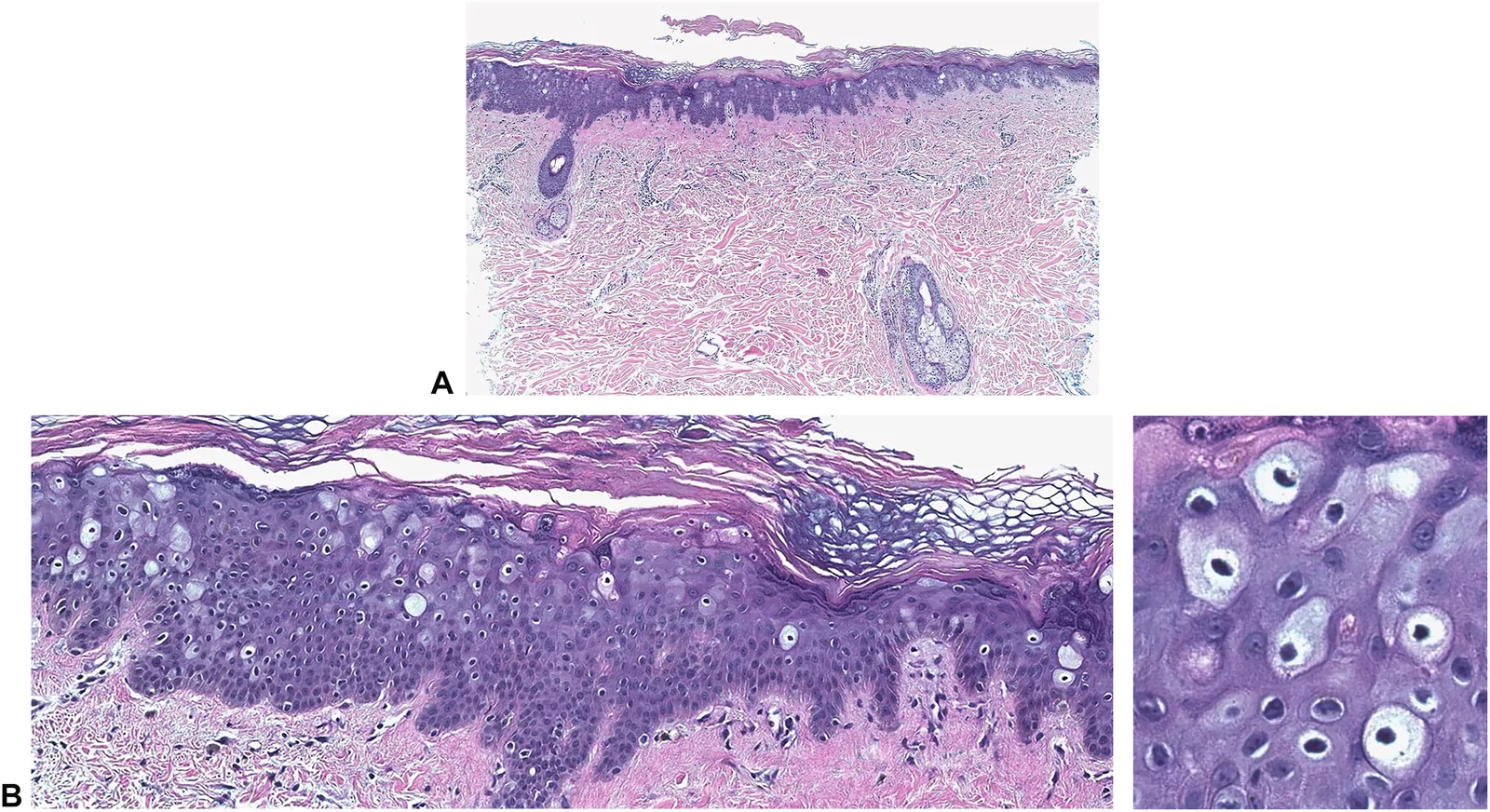
Representative hematoxylin and eosin-stained histopathologic images from a 3 mm punch biopsy from the abdomen (**A,** ×4; **B,** ×20; inset ×40).

On examination, the patient had 2 linear, parallel, thin, hyperpigmented patches located on either side of the abdominal midline, extending from the xiphoid process inferiorly to immediately above the umbilicus (Fig 1, *A*). Each patch contained discrete 1-2 mm mildly hyperkeratotic papules arranged in a repetitive and evenly spaced linear array (Fig 1, *B*).

A 3 mm punch biopsy showed subtle epidermal acanthosis and zones of compact hyperkeratosis admixed with basket-weave orthokeratosis without hypergranulosis or parakeratosis (Fig 2, *A*). In addition, the epidermis contained round and enlarged keratinocytes with pale cytoplasm and a pyknotic nucleus surrounded by a pale halo, scattered in a buckshot distribution (Fig 2, *B*). No scar was present.

**Question: What is the most likely diagnosis for this patient?**

**A.** Koebnerized psoriasis
**B.** Extramammary Paget disease
**C.** Pagetoid dyskeratosis
**D.** Squamous cell carcinoma in situ
**E.** Lichen simplex chronicus

## Answer discussion

The correct answer is **C**.

Pagetoid dyskeratosis (PD) is a benign, usually incidental, histopathologic change described in skin biopsies from numerous different anatomic locations, including but not limited to nevi, lentigines, acrochordons, fibrous papules, seborrheic keratoses, and milia.^1^ In contrast, PD has also been reported as a unique pathologic finding corresponding to the specific clinical setting of patchy macular hyperpigmentation of the hands and fingers, as well as the forehead.^2^^,^^3^ This case broadens the clinical context in which PD has been reported to occur.

Initially considered an artifact of sample processing, PD is now believed to be a reactive dyskeratotic process secondary to acceleration or prematurity of the keratinization process. As such, PD cells give rise to normal overlying orthokeratotic stratum corneum without acantholysis or parakeratosis.^1^ Histologic recognition of PD is important to prevent misdiagnosis of malignant neoplasms that contain pagetoid cells, such as squamous cell carcinoma in situ and extramammary Paget disease, and subsequent inappropriate overtreatment. PD cells are also distinct from koilocytic changes of human papillomavirus characterized by large vacuolated cells with small pyknotic nuclei surrounded by clear cytoplasm and usually located superficially in the epidermis. While our patient’s clinical presentation could support lichen simplex chronicus, this entity is not supported by the absence of marked acanthosis and hypergranulosis and presence of the distinctive pagetoid cells describe above. Psoriasis is also not supported by the absence of sharply demarcated erythematous plaques with silvery scale and absence histologically of all of the following: broad parakeratosis, regular acanthosis with elongated rete, hypogranulosis, thinning of the suprapapillary plates, dilated vessels in dermal papillae, and neutrophils in an inflammatory cell infiltrate.

PD is benign and treatment is not required. In the clinical setting of patchy hand/finger discoloration, PD resolved spontaneously over months to years, after skin biopsy, or after cessation of a known external cause of friction.^4^ In contrast, PD in this patient persisted for at least 18 months without spontaneous remission, did not respond to topical steroids, keratolytics, or topical retinoids but resolved after cryotherapy.

## Conflicts of interest

None disclosed.
